# A dyadic longitudinal analysis of parent-adolescent inflammation trends and the role of shared socioeconomic characteristics on family inflammation

**DOI:** 10.1016/j.bbih.2024.100767

**Published:** 2024-04-04

**Authors:** Sarah Rocha, Julienne E. Bower, Jessica J. Chiang, Steve W. Cole, Michael R. Irwin, Teresa Seeman, Andrew J. Fuligni

**Affiliations:** a University of California, Los Angeles, Department of Psychology, Los Angeles, CA, 90095, USA; b University of California, Los Angeles, Cousins Center for Psychoneuroimmunology, Los Angeles, CA, 90095, USA; c Georgetown University, Department of Psychology, Washington, D.C., 20057, USA; d University of California, Los Angeles, David Geffen School of Medicine, Department of Psychiatry and Biobehavioral Sciences, Los Angeles, CA, 90095, USA; e University of California, Los Angeles, David Geffen School of Medicine, Division of Geriatrics, Los Angeles, CA, 90095, USA

**Keywords:** Inflammation, Family dependency, Socioeconomic status, C-reactive protein, Dyadic modeling

## Abstract

The objective of the present study was to evaluate the interdependency of parent-adolescent inflammation trends across time and to examine whether shared family socioeconomic characteristics explained between-family differences in parents' and adolescents' risk for inflammation. A total of N = 348 families, consisting of one parent and one adolescent child, were followed every two years in a three-wave longitudinal study. Sociodemographic questionnaires were used to determine parental educational attainment and family income-to-needs ratio (INR). At each time point, parents and adolescents collected dried blood spot (DBS) samples that were assayed for circulating CRP and log-transformed prior to analysis by longitudinal dyadic models. Models revealed significant differences in parents' and adolescents' inflammation trends over time (b_int_ = - 0.13, p < 0.001). While parental CRP levels remained relatively stable across the study period, adolescent CRP increased by approximately 38% between study waves. Parents' average CRP levels were positively correlated with adolescents' average CRP (r = 0.32, p < 0.001), but parental change in CRP over time was not significantly related to change in adolescents' CRP over time. Family dyads with higher parental educational attainment had lower average CRP (b = −0.08, p = 0.01), but parental education did not predict change in dyads' inflammation over time. Study findings suggest that shared family socioeconomic characteristics contribute to baseline similarities in parents' and adolescents’ inflammation and potentially point to adolescence as a period of inflammatory change where youth may diverge from parental inflammation trends.

## 1 Introduction

Elevated inflammation is increasingly considered a marker of health risk among adult populations due to its implications in the pathogenesis of major diseases that contribute to mortality, most notably cardiovascular disease (Arnold et al., 2021). But inflammation has also become of interest to developmental researchers because of its potential to shape multiple dimensions of youth health with consequences for future disease risk. For example, elevated levels of inflammatory markers have been linked with adolescent depressive symptoms (Beurel et al., 2020), poor quality sleep (Hall et al., 2015; Park et al., 2020), and heightened cardiometabolic health risk (Herder et al., 2007; Lund et al., 2020; Syrenicz et al., 2006).

Both genetic factors (Kluft and de Maat, 2003) and shared environmental characteristics, such as family socioeconomic status (SES) (Pankow et al., 2001), suggest that adolescent inflammation trends would share similarities to parent inflammation patterns; however, there is a dearth of literature quantitatively assessing whether adolescents' longitudinal risk for inflammation is related to their parents' inflammation patterns. Information on the magnitude of family dependency in adolescent longitudinal inflammation trends can provide insight into the determinants of youth health risk from adolescence into young adulthood. Longitudinal studies of pro-inflammatory markers, such as C-reactive protein (CRP), have more commonly been conducted in adult populations (e.g., Lassale et al., 2019; Newman et al., 2016; Stevenson et al., 2018), with fewer studies examining longitudinal trends in adolescent inflammation (e.g., Beales et al., 2021; Chiang et al., 2019; Copeland et al., 2012), with none to the authors' knowledge quantifying the similarity of parent and adolescent longitudinal inflammation trends. Given this, there is lack of clarity regarding whether adolescents’ changes in inflammation across time are matched with inflammatory changes in their parents.

Researchers can take advantage of dyadic longitudinal modeling procedures to clarify the family dependency of parent and adolescent risk for inflammation across time. First, dyadic longitudinal models allow researchers to measure the interdependency of parents' and adolescents’ CRP outcomes by quantifying the correlation between their average inflammation levels, and the correlation of their longitudinal change across time. If parents and adolescents show strong family interdependency, this could suggest that shared family characteristics may explain the development of inflammatory risk across time. Next, dyadic models can examine whether a shared family characteristic, such as SES, is predictive of inflammation outcomes for parents and adolescents, and quantify whether the magnitude of this effect is significantly different between parents and adolescents. Family socioeconomic resources, such as household income and parental educational attainment, are shared resources that influence the daily lives of both parents and youth (Conger et al., 1992). Other experiences that can be shared within the family setting, such as parent-child conflict, major life stressors, abuse and maltreatment, similarly have established links with inflammation (Baumeister et al., 2016; Coelho et al., 2014; Miller et al., 2024; Miller and Chen, 2010). Family-SES presents itself as an opportune candidate for examining family dependency as it reflects a multitude of shared psychosocial (Adler and Snibbe, 2003) and environmental exposures (Evans and Kantrowitz, 2002), which may then prompt further investigation into the specific impacts of family process-oriented measures. Utilizing data from both parents and their adolescents offers multiple perspectives of the same socioeconomic resources and allows researchers to examine whether these resources impact parents and youth to the same degree.

Such information is valuable given potentially important developmental changes during the transition from adolescence to young adulthood that may change the influence of shared family characteristics on inflammation outcomes. During adolescence, youth become increasingly peer-oriented and begin spending less time with family and more time with peers outside the home (Somerville, 2013; Williams et al., 2002). Compared to younger children, adolescents are generally permitted greater autonomy over daily routines (e.g., sleep schedules, diet, exercise) that have been linked with inflammatory processes (Wärnberg et al., 2007). As adolescents begin transitioning to young adulthood and pursue employment or higher education, the socioeconomic resources of their family may become less salient and, accordingly, family-SES may not impact parent and adolescent inflammation to the same magnitude. Yet, the systemic nature of socioeconomic barriers suggest that family SES may continue to have lasting impacts on adolescent health. Youth who grow up in low income households are more likely to be exposed to environmental pollutants that cause inflammation (Mathiarasan and Hüls, 2021), live in neighborhoods with fewer green spaces that allow for outdoor physical activity (Wen et al., 2013), and have poorer access to healthy and affordable food (Beaulac et al., 2009). Hence, a dyadic assessment of parent-adolescent inflammation can clarify both the strength of family-dependency in longitudinal inflammation trends and examine whether family resources impact parent and adolescent inflammation trends to similar or differing magnitudes.

The present study utilized a longitudinal sample of N = 348 parent-adolescent dyads who were assessed for circulating levels of the pro-inflammatory marker, CRP, a total of three times across a four year study period. A prior paper based on this dataset found an inverse association between parental education and adolescent CRP during the first wave of the study (Chiang et al., 2015), and second paper found a positive increase in adolescence CRP across the four year period (Chiang et al., 2019). However, neither paper included parental data to compare parent and adolescent inflammation trends and assess differences in SES-inflammation associations between parents and adolescents. The main goals of the current study were as follows: first, we examined whether there was a significant difference in parents' and adolescents' baseline CRP levels and their longitudinal change in CRP across the study period. If significant differences were observed, separate longitudinal trends for parents and adolescents were modeled to characterize their magnitude of change across time. Next, we assessed the degree of family dependency in parent-adolescent CRP by assessing the correlation between parents' and adolescents' baseline CRP and the correlation between parents' and adolescents' longitudinal change in CRP. Finally, we examined whether family socioeconomic resources (i.e., parental educational attainment and family income-to-needs ratio) predicted between-family differences in baseline and longitudinal CRP. We further examined whether there was a significant difference in the influence of family-SES on parent vs adolescent CRP outcomes. Together, these questions can shed light on the degree to which family socioeconomic characteristics contribute to interdependency in parents' and adolescents’ risk for inflammation.

## 2 Methods

### 2.1 Participants and procedures

Participants were N = 350 parent-adolescent dyads who participated in a three-wave longitudinal study that ran from October 2011 to August 2016 in the Los Angeles area. Families were recruited from local high schools via flyers and classroom presentations. Those interested in participating in the study were contacted by phone and written consent was obtained in-person from the parent and the adolescent child during the first study visit. In two cases a grandparent participated in place of the parent; however, for simplicity, we refer to all guardians as “parents” throughout the manuscript. A total of 316 parent-adolescent dyads participated in the first wave of data collection (2011–2012), which took place when adolescents and parents were on average 16.4 years and 45.7 years, respectively. The sample was ethnically diverse: 42.0% of adolescents identified as Latinx, 30.3% as European American, 21.4% as Asian American, and 6.9% identified with another ethnicity or did not report ethnic identity (Table 1). Adolescents were 56.6% female, while their parents (generally mothers) were 90.9% female.

**Table 1:** Sample characteristics at first study entry (i.e., wave 1 or wave 2).

Family SES Characteristics	n (%)	Mean (SD)
Family INR		2.81 (2.16)
Below poverty line (<1)	63 (18)	
1 to 2	77 (22)	
2 to 3	47 (14)	
>3	119 (34)	
Unknown	42 (12)	
Parental Education		7.19 (1.87)
Elementary	16 (5)	
Junior High	49 (14)	
High School	206 (59)	
College	72 (21)	
Graduate School	3 (1)	
Unknown	2 (1)	

Participant Characteristics	Parents		Adolescents	
	n (%)	Mean (SD)	n (%)	Mean (SD)
Gender				
Female	317 (91)		198 (57)	
Male	29 (8)		149 (43)	
Unknown	2 (1)		1 (<1)	
Ethnicity				
Latinx	142 (41)		146 (42)	
Asian	77 (22)		74 (21)	
White	103 (29)		106 (30)	
Another	26 (7)		22 (6)	
Age (years)		45.7 (6.97)		16.4 (0.74)
BMI (kg/m^2^)		27.66 (6.87)		23.13 (4.99)
Frequent Smoker	38 (8)		1 (<1)	
Frequent Alcohol User	36 (10)		7 (2)	

In the second wave of data collection (2013–2014), 214 of the parent-adolescent dyads returned and 34 new families (age matched to the adolescent) were added to the sample to account for attrition. The final wave of data collection (2015–2016) occurred approximately four years after study onset, with 184 dyads participating. To encourage retention, parents and adolescents each were compensated $50 in wave 1, $75 in wave 2, and $120 in wave 3, and were additionally provided two movie passes per study wave. Across the four year study period, the sample became higher SES as dyads with greater parental education were more likely to complete all possible study waves *r* (346) = 0.13, p = 0.01, as were dyads with a higher income-to-needs ratio, *r* (340) = 0.17, p = <0.01. Among parents, study completion varied by ethnicity, *F* (3, 346) = 7.79, p < 0.001, with Asian parents participating in the fewest waves of data collection. Parent age at study entry was not related to study completion. Given the minimal gender variance among parents, we did not assess study completion differences by gender. Among adolescents, study completion also varied by ethnicity *F* (3, 346) = 5.56, p < 0.001), with Asian adolescents completing the fewest study waves. Female gender was positively related to adolescent study completion, *r* (347) = 0.11, p = 0.04. Given that adolescents were age matched at study entry, we did not assess completion differences by adolescent age.

### 2.2 Measures

#### 2.2.1 Socioeconomic status (SES)

*Parental Education*. Parents were asked to self-report their highest level of education and (if applicable) the highest level of education obtained by the adolescents' other parent (1 = some elementary school; 2 = completed elementary school; 3 = some junior-high school; 4 = completed junior-high school; 5 = some high school; 6 = graduated from high school; 7 = trade or vocational school; 8 = some college; 9 = graduated from college; 10 = some medical, law, or graduate school; 11 = graduated from medical, law, or graduate school). If information regarding the other parent's educational attainment was available, responses for both parents were averaged for one composite score of family parental educational attainment. Education reported during the initial study visit was used in all analyses due to low variation in educational attainment over time. On average, parents had completed the equivalent of trade or vocational school (see Table 1).

*Family Income-to-Needs Ratio.* At each study wave, parents reported total household income over the past year, including all money earned by family members who contributed to household expenses. Household size at each study wave was determined by the number of people parents reported currently living with them at that time. Family members or dependent adults not currently living in the household were not included in the household size. Each family's income-to-needs ratio (INR) was computed by dividing total reported household income over the U.S. Department of Health and Human Services' federal poverty threshold according to household size, such that an INR = 1 represented a family living at the poverty level. For waves one through three, the 2012, 2014 and 2016 guidelines were used, respectively. Across all waves, families had a median INR of 2.6, suggesting that the median family income was approximately 2–3 times the amount needed to afford basic needs (Table 1). Mean family INR across the three waves was used in multilevel analyses to examine between-family differences in CRP outcomes.

#### 2.2.2 C-reactive protein

Dyads provided dried blood spot (DBS) samples at each study visit to assess CRP levels. DBS sampling is a non-invasive alternative to venipuncture that has been well-validated for the assessment of CRP (D'Cruz et al., 2020; McDade et al., 2004). Parents' and adolescents' fingers were cleaned with alcohol and a sterile, disposable microlancet was used to puncture the skin. After wiping away the first drop of capillary blood, up to seven drops were allowed to fall onto a standardized filter paper. Blood spot samples were dried overnight and then stored at −80C prior to assay. Samples were shipped to the Laboratory for Human Biology Research at Northwestern University and assayed for circulating concentrations of CRP using high-sensitivity enzyme-linked immunosorbent assay with good precision, reliability, and high correlation with plasma CRP from venous blood (McDade et al., 2004). Samples were run in duplicate, and intra- and interassay coefficients of variation were 6.4% and 9.3%, respectively. Thirty-seven samples fell below the assay's detection limit of 0.03 mg/L and were subsequently set to the 0.03 detection threshold. Initial analyses showed that the sample was highly positively skewed, and so CRP values were natural log-transformed prior to analysis (Pietras and Goodman, 2013). On average, parents and adolescents provided 2.07 and 2.01 of the of the three possible DBS samples, respectively.

#### 2.2.3 Covariates

To account for the potential impact of recent illnesses on CRP levels, parents and adolescents were asked to self-report whether they had felt sick or unwell in the past 24 h prior to DBS collection and all models controlled for incidence of self-reported illness. Analyses of SES associations with inflammation analyses additionally controlled for participants' gender, ethnicity, and age, as these factors can potentially confound SES associations with health (Dowd et al., 2010; Muscatell et al., 2020). Given minimal fluctuations in reported gender and ethnicity, parents' and adolescents’ self-reported identities at study onset were used for all analyses. Descriptive statistics of participant demographics are reported in Table 1.

### 2.3 Analysis

The present analyses evaluated parents' and adolescents’ CRP trends across time and assessed whether family socioeconomic resources predicted family differences in CRP outcomes. The initial sample consisted of 350 dyads measured approximately every two years over three study waves. Individual observations were dropped if participants were missing CRP data at a given wave (n = 662) and in cases where an alternate parent provided data for a study wave (n = 8), resulting in an analytic sample of N = 1430 observations from 348 dyads across the three waves of the study.

Observations were analyzed using a series of two-level, multilevel models (MLMs) for longitudinal distinguishable dyadic data in SPSS statistical software (Version 28.0), which allow for the inclusion of individuals with missing timepoints (Snijders, 2011). The first level (L-1) represents variability due to within-person repeated measures for both adolescents and parents, and the second level (L-2) represents between-dyad variability across parents and across adolescents. Although the dataset has three levels of theoretical nested variation (i.e., study waves nested within persons nested within dyads), only the lower two levels show random variation (see Bolger and Laurenceau, 2013 for more details). Thus, dyad role (parent vs adolescent) was treated as a fixed effect, allowing free estimation of the correlation between parents' and adolescents’ intercepts and error at each time point (Bolger and Laurenceau, 2013; Kenny et al., 2006).

Our first dyadic model tested whether CRP trends were significantly different in magnitude between parents and adolescents. An effect coded dummy variable for dyad role (1 = parent, −1 = adolescent) was added to the model to examine the significance of the difference between parents' and adolescents’ CRP intercepts, or average CRP, controlling for incidence of participant illness (0 = healthy, 1 = ill). The dyad role variable was then multiplied by study wave (centered at the study midpoint) to assess the difference in the CRP time slope between parents and adolescents (see Table 2a). If these terms were significant—suggesting a significant difference in CRP outcomes between parents and adolescents—a second, two-intercept model was run to assess simple slope estimates of average CRP and CRP change across time for parents and adolescents. In this model, the dyad role variable was replaced with two dummy variables: *“*Parent*”* (1 = parent, 0 = adolescent) and *“*Adolescent*”* (1 = adolescent, 0 = parent) to obtain the separate estimates of the intercept, or average CRP, for both parents and adolescents. These dummy variables were then multiplied by study wave to obtain the separate time slope estimates for parents and adolescents (as shown in Table 3a).

**Table 2:** Multilevel estimates of log-transformed C-reactive protein for parent-adolescent dyads.

2A. Fixed effects	Estimate (SE)	t	p	95% CI
Intercept	−0.53 (0.05)	−10.34	<0.001	−0.63–−0.43
Dyad Role	0.40 (0.04)	9.85	<0.001	0.32–0.48
Time	0.19 (0.03)	5.47	<0.001	0.12–0.25
Dyad Role x Time	−0.13 (0.03)	−4.23	<0.001	−0.2–−0.07
Illness	0.76 (0.11)	6.88	<0.001	0.55–0.98
2B. Random effects	Estimate (SE)	z	p	95% CI
*i. Level-2 Variance*				
Adolescent Intercept	0.85 (0.11)	7.51	<0.001	0.66–1.11
Adolescent Slope	0.10 (0.08)	1.27	0.21	0.02–0.45
Parent Intercept	1.11 (0.11)	10.02	<0.001	0.92–1.35
Parent Slope	0.03 (0.04)	0.74	0.46	0.002–0.45
*ii. Level-2 Covariance*				
Parent, Adolescent Intercepts	0.31 (0.08)	3.82	0.001	0.15–0.47
Parent, Adolescent Slopes	0.03 (0.04)	0.82	0.41	−0.05–0.12
*iii. Level-1 Variance*				
Adolescent Residual	0.85 (0.09)	9.10	<0.001	0.69–1.06
Parent Residual	0.50 (0.05)	9.61	<0.001	0.41–0.61
*iv. Level-1 Covariance*				
Parent, Adolescent Residuals	−0.01 (0.08)	−0.17	0.13	−0.16–0.13

Note: The top panel (a) denotes the model's fixed effects while the bottom panel (b) denotes the model's random effects, or between-dyad variability around the fixed effects. Dyad role is effect coded (parent = 1, adolescent = −1) and represents the difference in average log-CRP between parents and adolescents. The dyad role × time interaction term reflects the difference in parents' and adolescents' change in CRP between study waves. SE = standard error.

**Table 3:** Multilevel “simple slope” estimates of log-transformed C-reactive protein for parents and adolescents.

3A. Fixed effects	Estimate (SE)	t	p	95% CI
Adolescent Intercept	−0.93 (0.07)	−14.24	<0.001	−1.06–−0.8
Adolescent Time Slope	0.32 (0.05)	6.09	<0.001	0.22–0.42
Parent Intercept	−0.13 (0.07)	−2.04	0.04	−0.26–−0.004
Parent Time Slope	0.05 (0.04)	1.36	0.17	−0.02–0.13
Illness	0.76 (0.11)	6.89	<0.001	0.55–0.98
3B. Random effects	Estimate (SE)	z	p	95% CI
*i. Level-2 Variance*				
Adolescent Intercept	0.85 (0.11)	7.51	<0.001	0.65–1.1
Adolescent Slope	0.09 (0.07)	1.24	0.22	0.02–0.45
Parent Intercept	1.11 (0.11)	10.03	<0.001	0.91–1.35
Parent Slope	0.03 (0.04)	0.71	0.48	0.002 - 0.48
*ii. Level-2 Covariance*				
Parent, Adolescent Intercepts	0.31 (0.08)	3.83	0.001	0.15–0.47
Parent, Adolescent Slopes	0.03 (0.04)	0.82	0.41	−0.05–0.12
*iii. Level-1 Variance*				
Adolescent Residual	0.85 (0.09)	9.10	<0.001	0.69–1.06
Parent Residual	0.50 (0.05)	9.61	<0.001	0.41–0.61
*iv. Level-1 Covariance*				
Parent, Adolescent Residuals	−0.01 (0.08)	−0.17	0.13	−0.16–0.13

Note: The top panel (a) denotes the model's fixed effects while the bottom panel (b) denotes the model's random effects, or between-dyad variability around the fixed effects. A two-intercept modeling approach provided simple slope estimates of CRP intercepts (i.e., average CRP) and CRP time slopes (i.e., longitudinal change in CRP) for both parents and adolescents. SE = standard error.

Both models allowed parents and adolescents to vary randomly in their average CRP and change in CRP across time. We specified the models' covariance matrix of random effects to provide estimates of the covariation between adolescents and parents' random CRP intercepts (i.e., average CRP), and covariation between their random CRP slopes (i.e., change over time). Given the likelihood of interdependence between parents and adolescents even after controlling for individual linear trends (Planalp et al., 2017), the model's level-1 residual variance was modeled using the dependent error covariance structure, which allows the covariance of adolescent and parents' residuals to be nonzero (Singer and Willett, 2003).

The next models assessed whether family socioeconomic resources were predictive of CRP levels and change in CRP overtime for parents and adolescents (see Table 4). First, we assessed the association of parental education (grand-mean centered) with average CRP and CRP change over time. The parental education term was then multiplied by dyad role to evaluate if the effect of parental education on average CRP was significantly different between parents and adolescents. Parental education was similarly multiplied by dyad role and study wave to assess if there was a significant difference between parents and adolescents in the effect of parent education on longitudinal CRP change. Potential confounding factors linked with inflammation: gender (effect coded, −1 = male, 1 = female), age (grand-mean centered), and ethnicity (effect coded such that each ethnicity is compared to Latinx and the intercept reflects the grand mean) were additionally added to the model to examine whether parental education predicted inflammation outcomes above and beyond these variables. Next, a separate model tested these same associations using mean family INR (grand-mean centered) as the measure of SES. Simple slope analyses estimating separate SES associations for parents and adolescents were performed only if the SES x dyad role interaction terms were significant. Given the complexity of the fixed effects in the SES models, we simplified the random effects portion of the models to allow parents and adolescents to vary only in their CRP intercepts (i.e., average CRP). A full table of the fixed and random estimates for the SES models is provided in the Supplement. The following presentation of results are based on recommendations for longitudinal dyadic models given by Bolger and Laurenceau (2013).

**Table 4:** Multilevel fixed effect estimates of log-transformed C-reactive protein for parent-adolescent dyads as a function of socioeconomic resources.

SES Variable	Fixed effects	Estimate (SE)	t	p	95% CI
A. Parent Education	Intercept	−0.61 (0.07)	−9.30	<0.001	−0.74–−0.48
	Dyad Role	0.58 (0.15)	3.99	<0.001	0.29–0.86
	Parent Education	−0.08 (0.03)	−2.83	0.01	−0.13–−0.02
	Parent Education * Dyad Role	0.01 (0.02)	0.39	0.70	−0.04–0.05
	Time	0.20 (0.04)	5.29	<0.001	0.13–0.28
	Time * Dyad Role	−0.14 (0.03)	−4.35	<0.001	−0.2–−0.08
	Time * Parent Education	0.02 (0.02)	1.28	0.20	−0.01–0.06
	Time * Parent Education * Dyad Role	−0.02 (0.02)	−1.39	0.16	−0.06–0.01
	Age	−0.01 (0.01)	−1.32	0.19	−0.03–0.01
	Female Gender	0.03 (0.05)	0.53	0.60	−0.08–0.13
	Asian	−0.82 (0.12)	−6.69	<0.001	−1.06–−0.58
	White	−0.12 (0.11)	−1.07	0.29	−0.35–0.10
	Another Ethnicity	−0.34 (0.19)	−1.83	0.07	−0.71–0.02
B. Family INR	Intercept	−0.63 (0.07)	−9.42	<0.001	−0.76–−0.50
	Dyad Role	0.56 (0.15)	3.87	<0.001	0.28–0.85
	Family INR	−0.04 (0.02)	−1.70	0.09	−0.08–0.01
	Family INR * Dyad Role	−0.03 (0.02)	−1.95	0.05	−0.07 – <0.001
	Time	0.20 (0.04)	5.31	<0.001	0.13–0.28
	Time * Dyad Role	−0.13 (0.03)	−4.12	<0.001	−0.2–−0.07
	Time * Family INR	0.01 (0.01)	0.27	0.79	−0.02–0.03
	Time * Family INR * Dyad Role	−0.02 (0.01)	−1.16	0.25	−0.04–0.01
	Age	−0.01 (0.01)	−1.13	0.26	−0.03–0.01
	Female Gender	0.03 (0.05)	0.55	0.58	−0.08–0.14
	Asian	−0.86 (0.13)	−6.86	<0.001	−1.11–−0.61
	White	−0.16 (0.12)	−1.38	0.17	−0.40–0.07
	Another Ethnicity	−0.42 (0.19)	−2.23	0.03	−0.79–−0.05

*Note*: The top panel displays fixed effect estimates of dyads' log-CRP as a function of parental educational attainment. Dyad role is effect coded (parent = 1, adolescent = −1) and represents the difference in average log-CRP between parents and adolescents. Hence, the parent education x dyad role interaction term reflects the influence of parental education on the difference in parents' and adolescents' average log-CRP. Similarly, the time x parental education x dyad role interaction term reflects the influence of parental education on the difference in parents' and adolescents' change in log-CRP between study waves. The bottom panel displays these same estimates as a function of family INR. For the models' random effects estimates, see Supplemental Materials. SE = standard error.

## 3 Results

**Fig. 1:**
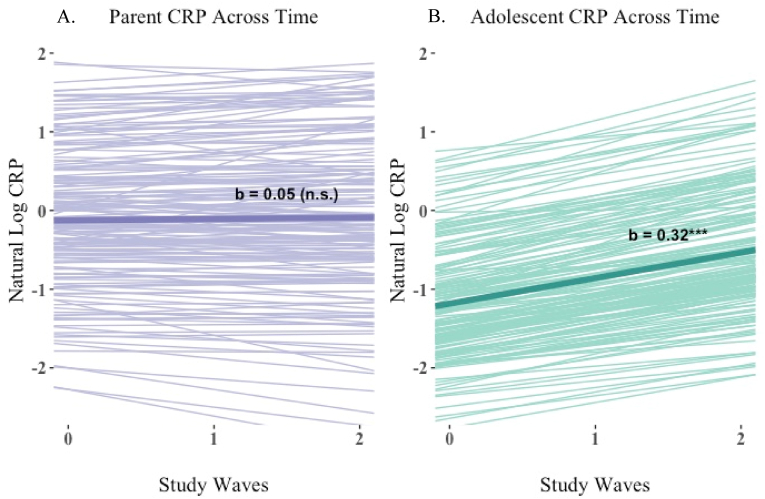
Multilevel estimates of log-CRP for parents and adolescents across the study period.

The first model assessed CRP trends for the full sample and estimated the magnitude of the difference in CRP outcomes between parents and adolescents. The average, or fixed components of the model are denoted in Table 2a. First, there was a significant effect of dyad role (b = 0.40, p < 0.001), suggesting that parents and adolescents had significantly different CRP concentrations at the study midpoint, controlling for illness. Similarly, the dyad role by time interaction term was also significant (b_int_ = - 0.13, p < 0.001), indicating that parents and adolescents significantly varied in their longitudinal change in CRP between study waves. Given this, a second model obtained separate simple slope estimates for parents and adolescents (Table 3a). As shown in Table 3a, the average CRP concentration for adolescents was −0.93 natural log units, corresponding to 0.39 mg/L in the original metric. The average CRP concentration for parents was −0.13 natural log units (i.e., 0.88 mg/L in the original metric), indicating that parents had higher CRP levels on average compared to adolescents. Next, looking at CRP time slopes, the average adolescent CRP increased by 38% between study waves, which differed significantly from zero (b = 0.32, p < 0.001). In contrast, we observed that parental CRP increased by 5.5% between study waves, which was not significantly greater than zero (b = 0.05, p = 0.17). These results are visualized in Fig. 1, which compares the model-estimated CRP trends for parents and adolescents across the study period.

Both models estimated equivalent random effects (i.e., the between-dyad variability around the fixed estimates), which are reported Table 2, Table 3b For brevity, random effect estimates from the first model are summarized here. As seen in Table 2b_ii_, adolescents' CRP intercepts significantly covaried with their parents' CRP intercepts (cov = 0.31, p < 0.001), corresponding to a correlation of r = 0.32 between parents' and adolescents' predicted average CRP. Thus, if a parent had higher average CRP compared to others in the sample, the model predicted that their adolescent also had a higher average CRP compared to others. In contrast, adolescents' longitudinal change (i.e., CRP time slope) did not significantly covary with parents' change in CRP over time (cov = 0.03, p = 0.41; Table 2b_ii_), suggesting that parents' and adolescents' change across time was not strongly related. Finally, as seen in Table 2b_iv_, adolescents' and parents’ residual variance was not significantly related after accounting for individual linear trends (cov = −0.01, p = 0.87). Thus, if an adolescent had higher than typical CRP at a given study wave, there was not significant evidence to suggest this was related to their parent having higher than typical CRP.

Next, we modeled family-SES associations with dyads' average and longitudinal CRP (controlling for gender, age, ethnicity, and illness) and evaluated whether the fixed effect of SES was significantly different between parents and adolescents (Table 4). The first of these models used parental educational attainment as the measure of family-SES. As seen in Table 4a, there was no significant difference in the effect of parental education on average (b_int_ = 0.01, p = 0.70) or longitudinal (b_int_ = −0.02, p = 0.16) CRP between parents and adolescents. However, for the sample as a whole, parental education was significantly associated with average CRP (b = −0.08, p = 0.01), such that a family dyad with one unit higher parental educational attainment (e.g., going from partial high school completion to high school graduate) was predicted to have an 8% lower average CRP concentration. Notably, parental educational attainment did not predict longitudinal change in dyads' CRP levels across the study period (b = 0.02, p = 0.20). The next model repeated these models using family-INR as the indicator of SES (Table 4b). We again observed no significant difference in the magnitude of the effect of INR between parents and adolescents for average (b_int_ = −0.03, p = 0.05) or longitudinal (b_int_ = −0.02, p = 0.25) CRP. Furthermore, family-INR did not predict dyads’ average (b = −0.04, p = 0.09) or longitudinal CRP (b = 0.01, p = 0.79), suggesting that family-INR may not be a strong predictor of differences in CRP levels between families. Random effect estimates for both SES models are reported in the Supplement.

## 4 Discussion

The present study evaluated the familial dependency of longitudinal CRP trends in a community sample of parent-adolescent dyads and examined the role of shared family socioeconomic variables on CRP outcomes. We found that family dependency was evidenced most strongly in parents' and adolescents' average—rather than longitudinal—inflammation outcomes. Longitudinal dyadic models indicated that parents' average CRP levels were positively correlated with adolescents' average CRP levels, such that parents with higher CRP concentrations tended to also have adolescents with higher CRP. In contrast, we did not observe a significant correlation between parents' and adolescents' change in CRP across time. This finding could be related to the significant differences in parents' and adolescents' longitudinal trends. Notably, adolescents demonstrated a 38% increase in CRP between each two-year study period whereas parental CRP remained relatively stable. Finally, we evaluated whether shared family-SES characteristics explained between-family differences in participants' CRP trends and whether the magnitude of this effect differed between parents and adolescents. We observed that higher parental education was related to lower CRP levels among dyads, and the strength of this effect did not significantly differ between parents and adolescents. In terms of longitudinal change, neither parental education nor family-INR predicted change in dyads’ CRP levels across time.

The observation that adolescents' baseline CRP concentrations were significantly correlated with their parents' CRP is consistent with a prior study comparing inflammatory markers between parents and children in healthy families (Haddy et al., 2003) and is further consistent with work suggesting that CRP levels are substantially heritable (Pankow et al., 2001). The degree to which adolescent longitudinal CRP trends map onto parent longitudinal patterns is less understood in the literature. In the present study, we found that there was no significant correlation between parents' change in CRP across the study period and adolescents' change in CRP. Although parents had significantly higher overall levels of CRP, their longitudinal change was not significantly different from zero across the four-year period, whereas adolescent CRP increased substantially across the same period. CRP concentrations are generally thought to increase with age (Ferrucci and Fabbri, 2018; Hutchinson et al., 2000); however, prior work suggests that there can be substantially heterogeneity in adults’ longitudinal CRP trends, with subgroups of individuals showing no change or decreasing CRP over time (Lassale et al., 2019; Stevenson et al., 2018). Hence, the insignificant change among parents observed in this study could be reflective of interindividual differences in longitudinal change, or alternatively, could suggest that a four-year window is insufficient to detect CRP changes in adult populations.

Importantly, the substantial increase in adolescent CRP observed in this study points to adolescence as a potentially important period to examine for the emergence of adult health risk. Developmental changes characteristic to adolescence may have contributed to the greater magnitude of inflammatory change among adolescents compared to parents. First, there are known changes to sleep behavior and circadian rhythms during adolescence that result in delays in bedtime and decreases in duration (Owens et al., 2014). In line with this, previous analyses of the present dataset found that youths’ actigraphy-scored sleep became shorter and more variable across the study period (Park et al., 2019). These sleep outcomes were furthermore associated with higher levels of CRP (Park et al., 2020), consistent with prior work linking sleep disturbance with pro-inflammatory signaling (Irwin, 2019). These links highlight sleep as a potential biobehavioral driver of inflammatory change during adolescence; however, the lack of sleep actigraphy data for the parent participants limited our ability to test whether sleep explained the differential magnitude of CRP change between parents and adolescents. Other potential developmental changes that may underlie increases in inflammation across adolescence include normative reductions in exercise (Corder et al., 2015) and increases in adiposity (Sanyaolu et al., 2019). Prior longitudinal studies have observed that increasing or “stable-high” BMI trajectories from adolescence to adulthood are related to elevated CRP levels (Attard et al., 2013; Beales et al., 2021). Furthermore, previous literature suggests that early risk signs for chronic inflammatory diseases, such as cardiovascular disease, may first appear during adolescence and young adulthood (Shay et al., 2013). Together, these findings present adolescence as a period of marked changes in CRP in which youth begin to diverge from parental inflammation trends. The results of this study may inform future intervention efforts—highlighting adolescence as a potential target for early risk detection and opportunity to modify behaviors that drive pro-inflammatory trajectories.

Study findings also may indicate that baseline similarities in parent-adolescent CRP levels are related to shared family socioeconomic resources. Consistent with prior work in both adults (Muscatell et al., 2020) and adolescents (Pietras and Goodman, 2013; Saxton et al., 2011), higher parental education was linked with lower average CRP levels. Interestingly, we found that the magnitude of this effect was not significantly different between parents and adolescents, despite parental education being a more “proximal” measure of SES to parents. Furthermore, another shared socioeconomic resource, family INR, did not predict differences in baseline CRP between families. The present findings could indicate that parental education is particularly important measure of socioeconomic status for predicting inflammatory risk in families. Prior work in adults suggests that higher educational attainment is linked with health-promoting behaviors that reduce risk for inflammation (Petrovic et al., 2018). Parents with higher educational attainment in turn may be more likely to engage in preventative health parenting practices with their children, such as encouragement of youths' daily exercise (Kantomaa et al., 2007; Prickett and Augustine, 2016), nutritious eating behaviors (Glozah and Pevalin, 2015; Okamoto, 2021), and consistent sleep routines (Lee et al., 2019). However, parental education did not predict longitudinal change in CRP over the course of the study. While this finding is inconsistent with previous studies that observed longitudinal associations of SES with inflammation across the lifespan (Pollitt et al., 2007; Stringhini et al., 2013) it contributes to an overall mixed literature regarding the predictive ability of childhood-SES on inflammation into adulthood after accounting for adult-SES (Milaniak and Jaffee, 2019). In the context of the current study, the inability of family-SES to predict longitudinal CRP may be one reason why parents' and adolescents' longitudinal trends were unrelated. Hence, parental education may be important for establishing health behaviors that contribute to families' baseline inflammation levels, but its’ predictive power may diminish as adolescents enter young adulthood.

The present study had several strengths. First, the findings offer novel insights into the family dependency of parent-adolescent longitudinal inflammation outcomes, the magnitude of which, to the authors’ knowledge, has not previously been explored. The longitudinal CRP data further provided an opportunity to characterize both parent and adolescent longitudinal inflammation trends—which pointed to adolescence as a potential period of substantial change in inflammation with implications for future adult health. Next, the current study included multiple SES measures, which allowed us to quantify the impact of the socioeconomic positioning of the family on parents and adolescents and compare the magnitude of the association for each. Parental education was identified as a particularly influential variable in predicting both parent and adolescent information, and this information can be used by researchers looking to identify risk factors for family inflammation.

Limitations of the present study include the data being observational in nature and thus unable to support causal conclusions regarding parents' influence on youth inflammation or the effect of SES on inflammation. While the longitudinal nature of the study brought clarity to the potential time effects of SES, the relatively short time span (approximately four years) limited power to assess CRP change across time or examine the effects of socioeconomic mobility on CRP outcomes. Further, we did not measure youth during early adolescence or obtain information regarding pubertal development, preventing us from assessing developmental interactions with SES that may contribute to inflammatory change. The present study also did not assess other experiences within the family setting such as conflict, stressful events, and maltreatment, that may have contributed to the observed baseline similarities in parent-adolescent CRP. Finally, this study focused on characterizing parent-adolescent inflammation trends and did not assess chronic inflammatory diseases or the clinical significance of CRP levels, hence, the findings’ clinical significance for long-term adult health is limited.

## 5 Conclusion

The present study provided evidence that shared family characteristics influence baseline inflammation outcomes for parents and adolescents, and that parental educational attainment represents one key determinant of those shared effects. Notable differences in the longitudinal stability of CRP levels between adolescents and parents were detected, with adolescents showing a greater increase in CRP levels over time compared to parents. Results from this study point to adolescence as a potentially opportune period to examine for and intervene against the emergence of inflammatory health risk. Future research can identify shared environmental, behavioral, and psychological factors that may explain interdependency in families’ risk for inflammation across time.

## Source Funding

The study was supported by funding from the Eunice Kennedy Shriver National Institute of Child Health and Human Development (R01-HD062547); the University of California, Los Angeles California Center for Population Research, which was supported by the National Institute of Child Health and Human Development (R24-HD041022); and the University of California, Los Angeles Older Americans Independence Center, which was supported by the National Institute of Aging (P30-AG017265 and P30-AG028748). The content does not necessarily represent the official views of the National Institutes of Health and its separate institutes.

## CRediT authorship contribution statement

**Sarah Rocha:** Writing – review & editing, Writing – original draft, Visualization, Formal analysis, Data curation, Conceptualization. **Julienne E. Bower:** Writing – review & editing, Investigation, Funding acquisition. **Jessica J. Chiang:** Writing – review & editing, Investigation, Data curation. **Steve W. Cole:** Writing – review & editing, Investigation, Funding acquisition. **Michael R. Irwin:** Writing – review & editing, Investigation, Funding acquisition. **Teresa Seeman:** Writing – review & editing, Investigation, Funding acquisition. **Andrew J. Fuligni:** Writing – review & editing, Supervision, Resources, Investigation, Funding acquisition.

## Declaration of competing interest

None.

## Data Availability

Data will be made available on request.

## References

[bib1] Adler N.E., Snibbe A.C. (2003). The role of psychosocial processes in explaining the gradient between socioeconomic status and health. Curr. Dir. Psychol. Sci..

[bib2] Arnold N., Lechner K., Waldeyer C., Shapiro M.D., Koenig W. (2021). Inflammation and cardiovascular disease: the future. Eur. Cardiol..

[bib3] Attard S.M., Herring A.H., Howard A.G., Gordon-Larsen P. (2013). Longitudinal trajectories of BMI and cardiovascular disease risk: the national longitudinal study of adolescent health. Obesity.

[bib4] Baumeister D., Akhtar R., Ciufolini S., Pariante C.M., Mondelli V. (2016). Childhood trauma and adulthood inflammation: a meta-analysis of peripheral C-reactive protein, interleukin-6 and tumour necrosis factor-α. Mol. Psychiatr..

[bib5] Beales D., Beynon A., Jacques A., Smith A., Cicuttini F., Straker L. (2021). Insight into the longitudinal relationship between chronic subclinical inflammation and obesity from adolescence to early adulthood: a dual trajectory analysis. Inflamm. Res.: Official Journal of the European Histamine Research Society ... [et Al..

[bib6] Beaulac J., Kristjansson E., Cummins S. (2009). A systematic review of food deserts, 1966-2007. Prev. Chronic Dis..

[bib7] Beurel E., Toups M., Nemeroff C.B. (2020). The bidirectional relationship of depression and inflammation: double trouble. Neuron.

[bib8] Bolger N., Laurenceau J.-P. (2013).

[bib9] Chiang J.J., Bower J.E., Almeida D.M., Irwin M.R., Seeman T.E., Fuligni A.J. (2015). Socioeconomic status, daily affective and social experiences, and inflammation during adolescence. Psychosom. Med..

[bib10] Chiang J.J., Park H., Almeida D.M., Bower J.E., Cole S.W., Irwin M.R., McCreath H., Seeman T.E., Fuligni A.J. (2019). Psychosocial stress and C-reactive protein from mid-adolescence to young adulthood. Health Psychol..

[bib11] Coelho R., Viola T.W., Walss-Bass C., Brietzke E., Grassi-Oliveira R. (2014). Childhood maltreatment and inflammatory markers: a systematic review. Acta Psychiatr. Scand..

[bib12] Conger R.D., Conger K.J., Elder G.H., Lorenz F.O., Simons R.L., Whitbeck L.B. (1992). A family process model of economic hardship and adjustment of early adolescent boys. Child Dev..

[bib13] Copeland W.E., Shanahan L., Worthman C., Angold A., Costello E.J. (2012). Cumulative depression episodes predict later C-reactive protein levels: a prospective analysis. Biol. Psychiatr..

[bib14] Corder K., Sharp S.J., Atkin A.J., Griffin S.J., Jones A.P., Ekelund U., van Sluijs E.M.F. (2015). Change in objectively measured physical activity during the transition to adolescence. Br. J. Sports Med..

[bib15] D'Cruz L.G., McEleney K.G., Cochrane C., Tan K.B.C., Shukla P., Gardiner P.V., Small D., Zhang S.-D., Gibson D.S. (2020). Assessment of a dried blood spot C-reactive protein method to identify disease flares in rheumatoid arthritis patients. Sci. Rep..

[bib16] Dowd J.B., Zajacova A., Aiello A.E. (2010). Predictors of inflammation in U.S. Children aged 3–16 years. Am. J. Prev. Med..

[bib17] Evans G.W., Kantrowitz E. (2002). Socioeconomic status and health: the potential role of environmental risk exposure. Annu. Rev. Publ. Health.

[bib18] Ferrucci L., Fabbri E. (2018). Inflammageing: chronic inflammation in ageing, cardiovascular disease, and frailty. Nat. Rev. Cardiol..

[bib19] Glozah F.N., Pevalin D.J. (2015). Perceived social support and parental education as determinants of adolescents' physical activity and eating behaviour: a cross-sectional survey. Int. J. Adolesc. Med. Health.

[bib20] Haddy N., Sass C., Droesch S., Zaiou M., Siest G., Ponthieux A., Lambert D., Visvikis S. (2003). IL-6, TNF-α and atherosclerosis risk indicators in a healthy family population: the STANISLAS cohort. Atherosclerosis.

[bib21] Hall M.H., Lee L., Matthews K.A. (2015). Sleep duration during the school week is associated with C-reactive protein risk groups in healthy adolescents. Sleep Med..

[bib22] Herder C., Schneitler S., Rathmann W., Haastert B., Schneitler H., Winkler H., Bredahl R., Hahnloser E., Martin S. (2007). Low-Grade inflammation, obesity, and insulin resistance in adolescents. J. Clin. Endocrinol. Metabol..

[bib23] Hutchinson W.L., Koenig W., Fröhlich M., Sund M., Lowe G.D.O., Pepys M.B. (2000). Immunoradiometric assay of circulating C-reactive protein: age-related values in the adult general population. Clin. Chem..

[bib24] Irwin M.R. (2019). Sleep and inflammation: partners in sickness and in health. Nat. Rev. Immunol..

[bib25] Kantomaa M.T., Tammelin T.H., Näyhä S., Taanila A.M. (2007). Adolescents' physical activity in relation to family income and parents' education. Prev. Med..

[bib26] Kenny D.A., Kashy D.A., Cook W.L. (2006).

[bib27] Kluft C., de Maat M.P.M. (2003). Genetics of C-reactive protein. Arterioscler. Thromb. Vasc. Biol..

[bib28] Lassale C., Batty G.D., Steptoe A., Cadar D., Akbaraly T.N., Kivimäki M., Zaninotto P. (2019). Association of 10-year C-reactive protein trajectories with markers of healthy aging: findings from the English longitudinal study of aging. J. Gerontol.: Series A.

[bib29] Lee S., Hale L., Berger L.M., Buxton O.M. (2019). Maternal perceived work schedule flexibility predicts child sleep mediated by bedtime routines. J. Child Fam. Stud..

[bib30] Lund M.A.V., Thostrup A.H., Frithioff-Bøjsøe C., Lausten-Thomsen U., Hedley P.L., Pedersen O., Christiansen M., Hansen T., Holm J.-C. (2020). Low-grade inflammation independently associates with cardiometabolic risk in children with overweight/obesity. Nutr. Metabol. Cardiovasc. Dis..

[bib31] Mathiarasan S., Hüls A. (2021). Impact of environmental injustice on children's health—interaction between air pollution and socioeconomic status. Int. J. Environ. Res. Publ. Health.

[bib32] McDade T.W., Burhop J., Dohnal J. (2004). High-sensitivity enzyme immunoassay for C-reactive protein in dried blood spots. Clin. Chem..

[bib33] Milaniak I., Jaffee S.R. (2019). Childhood socioeconomic status and inflammation: a systematic review and meta-analysis. Brain Behav. Immun..

[bib34] Miller G.E., Carroll A.L., Armstrong C.C., Craske M.G., Zinbarg R.E., Bookheimer S.Y., Ka-Yi Chat I., Vinograd M., Young K.S., Nusslock R. (2024). Major stress in early childhood strengthens the association between peripheral inflammatory activity and corticostriatal responsivity to reward. Brain Behav. Immun..

[bib35] Miller G.E., Chen E. (2010). Harsh family climate in early life presages the emergence of a proinflammatory phenotype in adolescence. Psychol. Sci..

[bib36] Muscatell K.A., Brosso S.N., Humphreys K.L. (2020). Socioeconomic status and inflammation: a meta-analysis. Mol. Psychiatr..

[bib37] Newman A.B., Sanders J.L., Kizer J.R., Boudreau R.M., Odden M.C., Zeki Al Hazzouri A., Arnold A.M. (2016). Trajectories of function and biomarkers with age: the CHS all stars study. Int. J. Epidemiol..

[bib38] Okamoto S. (2021). Parental socioeconomic status and adolescent health in Japan. Sci. Rep..

[bib39] Owens J., Au R., Carskadon M., Millman R., Wolfson A., Braverman P.K., Adelman W.P., Breuner C.C., Levine D.A., Marcell A.V., Murray P.J., O'Brien R.F. (2014). Insufficient sleep in adolescents and young adults: an update on causes and consequences. Pediatrics.

[bib40] Pankow J.S., Folsom A.R., Cushman M., Borecki I.B., Hopkins P.N., Eckfeldt J.H., Tracy R.P. (2001). Familial and genetic determinants of systemic markers of inflammation: the NHLBI family heart study. Atherosclerosis.

[bib41] Park H., Chiang J.J., Bower J.E., Irwin M.R., Almeida D.M., Seeman T.E., McCreath H., Fuligni A.J. (2020). Sleep and inflammation during adolescents' transition to young adulthood. J. Adolesc. Health.

[bib42] Park H., Chiang J.J., Irwin M.R., Bower J.E., McCreath H., Fuligni A.J. (2019). Developmental trends in sleep during adolescents' transition to young adulthood. Sleep Med..

[bib43] Petrovic D., de Mestral C., Bochud M., Bartley M., Kivimäki M., Vineis P., Mackenbach J., Stringhini S. (2018). The contribution of health behaviors to socioeconomic inequalities in health: a systematic review. Prev. Med..

[bib44] Pietras S.A., Goodman E. (2013). Socioeconomic status gradients in inflammation in adolescence. Psychosom. Med..

[bib45] Planalp E.M., Du H., Braungart-Rieker J.M., Wang L. (2017). Growth curve modeling to studying change: a comparison of approaches using longitudinal dyadic data with distinguishable dyads. Struct. Equ. Model.: A Multidiscip. J..

[bib46] Pollitt R.A., Kaufman J.S., Rose K.M., Diez-Roux A.V., Zeng D., Heiss G. (2007). Early-life and adult socioeconomic status and inflammatory risk markers in adulthood. Eur. J. Epidemiol..

[bib47] Prickett K.C., Augustine J.M. (2016). Maternal education and investments in children's health. J. Marriage Fam..

[bib48] Sanyaolu A., Okorie C., Qi X., Locke J., Rehman S. (2019). Childhood and adolescent obesity in the United States: a public health concern. Global Pediatric Health.

[bib49] Saxton K.B., John-Henderson N., Reid M.W., Francis D.D. (2011). The social environment and IL-6 in rats and humans. Brain Behav. Immun..

[bib50] Shay C.M., Ning H., Daniels S.R., Rooks C.R., Gidding S.S., Lloyd-Jones D.M. (2013). Status of cardiovascular health in US adolescents. Circulation.

[bib51] Singer J.D., Willett J.B. (2003).

[bib52] Snijders T.A.B. (2011).

[bib53] Somerville L.H. (2013). The teenage brain: sensitivity to social evaluation. Curr. Dir. Psychol. Sci..

[bib54] Stevenson A.J., McCartney D.L., Harris S.E., Taylor A.M., Redmond P., Starr J.M., Zhang Q., McRae A.F., Wray N.R., Spires-Jones T.L., McColl B.W., McIntosh A.M., Deary I.J., Marioni R.E. (2018). Trajectories of inflammatory biomarkers over the eighth decade and their associations with immune cell profiles and epigenetic ageing. Clin. Epigenet..

[bib55] Stringhini S., Batty G.D., Bovet P., Shipley M.J., Marmot M.G., Kumari M., Tabak A.G., Kivimäki M. (2013). Association of lifecourse socioeconomic status with chronic inflammation and type 2 diabetes risk: the Whitehall II prospective cohort study. PLoS Med..

[bib56] Syrenicz A., Garanty-Bogacka B., Syrenicz M., Gębala A., Walczak M. (2006).

[bib57] Wärnberg J., Nova E., Romeo J., Moreno L.A., Sjöström M., Marcos A. (2007). Lifestyle-related determinants of inflammation in adolescence. Br. J. Nutr..

[bib58] Wen M., Zhang X., Harris C.D., Holt J.B., Croft J.B. (2013). Spatial disparities in the distribution of parks and green spaces in the USA. Ann. Behav. Med..

[bib59] Williams P.G., Holmbeck G.N., Greenley R.N. (2002). Adolescent health psychology. J. Consult. Clin. Psychol..

