# Supplementary material for: A dyadic longitudinal analysis of parent-adolescent inflammation trends and the role of shared socioeconomic characteristics on family inflammation
Source: Brain Behav Immun Health. 2024 Apr 4;38:100767. doi: 10.1016/j.bbih.2024.100767 (PMC11021828; doi:10.1016/j.bbih.2024.100767)
Supplement: Multimedia component 1 [file mmc1.docx]

Supplemental Tables

Supplemental Table 1. Multilevel estimates of C-reactive protein for parent-adolescent dyads as a function of parental educational attainment

| 1A. Fixed effects | Estimate (SE) | t | p | 95% CI |
| --- | --- | --- | --- | --- |
|  |  |  |  |  |
| Intercept | -0.61 (0.07) | -9.30 | <.001 | -0.74 – -0.48 |
| Dyad Role | 0.58 (0.15) | 3.99 | <.001 | 0.29 – 0.86 |
| Parent Education | -0.08 (0.03) | -2.83 | .01 | -0.13 – -0.02 |
| Parent Education * Dyad Role | 0.01 (0.02) | 0.39 | .70 | -0.04 – 0.05 |
| Time | 0.20 (0.04) | 5.29 | <.001 | 0.13 – 0.28 |
| Time * Dyad Role | -0.14 (0.03) | -4.35 | <.001 | -0.2 – -0.08 |
| Time * Parent Education | 0.02 (0.02) | 1.28 | .20 | -0.01 – 0.06 |
| Time * Parent Education * Dyad Role | -0.02 (0.02) | -1.39 | .16 | -0.06 – 0.01 |
| Age | -0.01 (0.01) | -1.32 | .19 | -0.03 – 0.01 |
| Female Gender | 0.03 (0.05) | 0.53 | .60 | -0.08 – 0.13 |
| Asian | -0.82 (0.12) | -6.69 | <.001 | -1.06 – -0.58 |
| White | -0.12 (0.11) | -1.07 | .29 | -0.35 – 0.10 |
| Another Ethnicity | -0.34 (0.19) | -1.83 | .07 | -0.71 – 0.02 |
| Illness | 0.72 (0.11) | 6.33 | <.001 | 0.50 – 0.94 |
|  |  |  |  |  |
| 1B. Random effects | Estimate (SE) | z | p | 95% CI |
|  |  |  |  |  |
| *i. Level-2 Variance* |  |  |  |  |
| Adolescent Intercept | 0.68 (0.10) | 6.97 | <.001 | 0.52 – 0.91 |
| Parent Intercept | 0.87 (0.09) | 9.33 | <.001 | 0.71 – 1.07 |
|  |  |  |  |  |
| *ii. Level-2 Covariance* |  |  |  |  |
| Parent, Adolescent Intercept | 0.14 (0.07) | 2.00 | .05 | 0.003 – 0.28 |
|  |  |  |  |  |
| *iii. Level-1 Variance* |  |  |  |  |
| Adolescent Residual | 0.95 (0.07) | 13.15 | <.001 | 0.82 – 1.10 |
| Parent Residual | 0.52 (0.04) | 12.94 | <.001 | 0.45 – 0.61 |
|  |  |  |  |  |
| *iv. Level-1 Covariance* |  |  |  |  |
| Parent, Adolescent Residual | 0.02 (0.06) | 0.35 | .13 | -0.09 – 0.14 |
|  |  |  |  |  |

*Table 1 Note*: The top panel (a) denotes the model’s fixed effects while the bottom panel (b) denotes the model’s random effects, or between-dyad variability around the fixed effects. Dyad role is effect coded (parent = 1, adolescent = -1) and represents the difference in average log-CRP between parents and adolescents. Hence, the parent education x dyad role interaction term reflects the influence of parental education on the difference in parents’ and adolescents’ average log-CRP. Similarly, the time x parental education x dyad role interaction term reflects the influence of parental education on the difference in parents’ and adolescents’ change in log-CRP between study waves.

Supplemental Table 2. Multilevel estimates of C-reactive protein for parent-adolescent dyads as a function of family income-to-needs ratio (INR)

| 2A. Fixed effects | Estimate (SE) | t | p | 95% CI |
| --- | --- | --- | --- | --- |
|  |  |  |  |  |
| Intercept | -0.63 (0.07) | -9.42 | <.001 | -0.76 – -0.5 |
| Dyad Role | 0.56 (0.15) | 3.87 | <.001 | 0.28 – 0.85 |
| Family INR | -0.04 (0.02) | -1.70 | .09 | -0.08 – 0.01 |
| Family INR * Dyad Role | -0.03 (0.02) | -1.95 | .05 | -0.07 – <.001 |
| Time | 0.204 (0.04) | 5.31 | <.001 | 0.13 – 0.28 |
| Time * Dyad Role | -0.13 (0.03) | -4.12 | <.001 | -0.2 – -0.07 |
| Time * Family INR | 0.01 (0.01) | 0.27 | .79 | -0.02 – 0.03 |
| Time * Family INR * Dyad Role | -0.02 (0.01) | -1.16 | .25 | -0.04 – 0.01 |
| Age | -0.01 (0.01) | -1.13 | .26 | -0.03 – 0.01 |
| Female Gender | 0.03 (0.05) | 0.55 | .58 | -0.08 – 0.14 |
| Asian | -0.86 (0.13) | -6.86 | <.001 | -1.11 – -0.61 |
| White | -0.16 (0.12) | -1.38 | .17 | -0.399 – 0.07 |
| Another Ethnicity | -0.42 (0.19) | -2.23 | .03 | -0.79 – -0.05 |
| Illness | 0.73 (0.11) | 6.36 | <.001 | 0.502 – 0.95 |
|  |  |  |  |  |
| 2B. Random effects | Estimate (SE) | z | p | 95% CI |
|  |  |  |  |  |
| *i. Level-2 Variance* |  |  |  |  |
| Adolescent Intercept | 0.73 (0.1) | 7.17 | <.001 | 0.55 – 0.96 |
| Parent Intercept | 0.86 (0.09) | 9.20 | <.001 | 0.69 – 1.06 |
|  |  |  |  |  |
| *ii. Level-2 Covariance* |  |  |  |  |
| Parent, Adolescent Intercept | 0.16 (0.07) | 2.26 | .02 | 0.02 – 0.3 |
|  |  |  |  |  |
| *iii. Level-1 Variance* |  |  |  |  |
| Adolescent Residual | 0.95 (0.07) | 13.18 | <.001 | 0.82 – 1.10 |
| Parent Residual | 0.52 (0.04) | 12.90 | <.001 | 0.45 – 0.61 |
|  |  |  |  |  |
| *iv. Level-1 Covariance* |  |  |  |  |
| Parent, Adolescent Residual | 0.02 (0.06) | 0.34 | .13 | -0.1 – 0.13 |
|  |  |  |  |  |

*Table 2 Note*: The top panel (a) denotes the model’s fixed effects while the bottom panel (b) denotes the model’s random effects, or between-dyad variability around the fixed effects. Dyad role is effect coded (parent = 1, adolescent = -1) and represents the difference in average log-CRP between parents and adolescents. Hence, the family INR x dyad role interaction term reflects the influence of family INR on the difference in parents’ and adolescents’ average log-CRP. Similarly, the time x family INR x dyad role interaction term reflects the influence of family INR on the difference in parents’ and adolescents’ change in log-CRP between study waves.
